# Supplementary material for: Rhesus macaques self-curing from a schistosome infection can display complete immunity to challenge
Source: Nat Commun. 2021 Oct 26;12:6181. doi: 10.1038/s41467-021-26497-0 (PMC8548296; doi:10.1038/s41467-021-26497-0)
Supplement: Supplementary file 1 — Supplementary Information [file 41467_2021_26497_MOESM1_ESM.pdf]

Rhesus macaques self-curing from a schistosome infection can display complete immunity to challenge

Amaral *et al*.

Supplementary Information

a

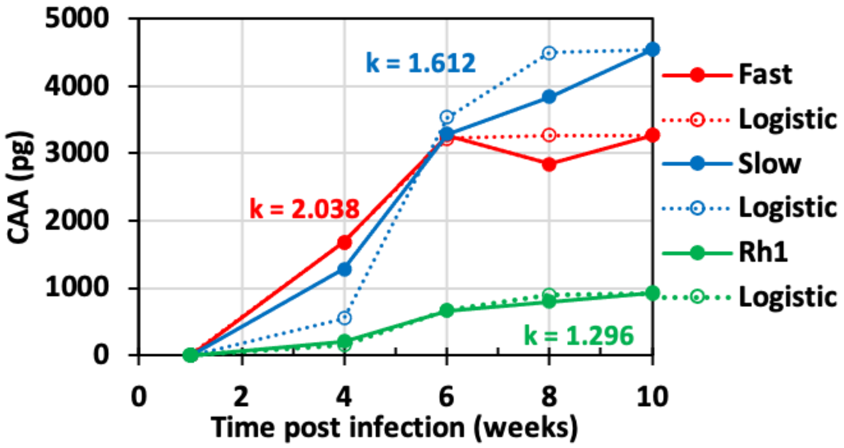

b

Logistic formula, $P_t = P_0M/P_0+(M-P_0)exp^{-kt}$
Where:
P_0 = CAA at wk1 = 1pg
M = maximum CAA, user defined from data
k is the rate constant, obtained using Solver in Excel

c

Groups	k
All rhesus - Rh1	1.914
Fast responders	2.038
Slow responders	1.612
Rh1	1.296

Supplementary Fig. 1. The establishment phase can be treated as a logistic growth process. a Mean circulating anodic antigen (CAA) values for Fast (red) and Slow (blue) responder groups plus Rh1 (green), over the first 10 weeks post-primary infection (solid circles and lines). The values predicted by the logistic equation fitted to the raw data (open circles and dotted lines). **b** The logistic equation with parameters defined to permit calculation of k, the rate constant for each data set. **c** Values for k in the logistic equation fitted to CAA data during growth phase. The greater the value of k the quicker the maximum is reached.

	Wk1pc		Peak CAA post challenge			Wk20pc	
	CAA	**Adult W Eq**	CAA	Week	**Adult W Eq**	CAA	**Adult W Eq**
**Rh2**	0	**0**	35	4	**3**	0	**0**
**Rh3**	5	**0**	52	4	**4**	6	**0**
**Rh4**	11	**1**	45	10	**3**	17	**1**
**Rh9**	48	**4**	64	4	**5**	36	**3**
**Rh7**	38	**3**	101	4	**7**	13	**1**
**Rh12**	23	**2**	143	4	**11**	35	**3**
**Rh1**	315	**23**	798	4	**59**	108	**8**
**Rh5**	134	**10**	342	4	**25**	76	**6**
**Rh6**	310	**23**	661	8	**49**	245	**18**
**Rh8**	152	**11**	429	4	**32**	275	**20**
**Rh11**	114	**8**	326	4	**24**	184	**14**
	Total =	**84**		Total =	**220**	Total =	**73**

Supplementary Fig. 2. Summary of post challenge circulating anodic antigen (CAA) data. The data underlying Fig. 4 are tabulated at three points post challenge. Wk1pc represents the absolute low point for CAA in each animal before blood feeding by challenge worms had begun, together with a prediction of the size of the residual worm population in adult worm equivalents (Adult W Eq), based on Fallon *et al*. (Supplementary ref. 1). Two animals were predicted to have cleared their primary population and four others virtually so. The peak CAA post challenge represents the maximum level attained in that animal and its week in the next column, followed by predicted adult worm equivalents. The CAA values at the Wk20pc termination of the experiment reveal that two animals had no predicted worm equivalents, and four other animals had three or less worms, the same six animals that had virtually cleared the primary burden. In the first column rhesus macaque groups are designated as follows: Fast, red; Slow, blue; Intermediate, black; Outlier Rh1, green.

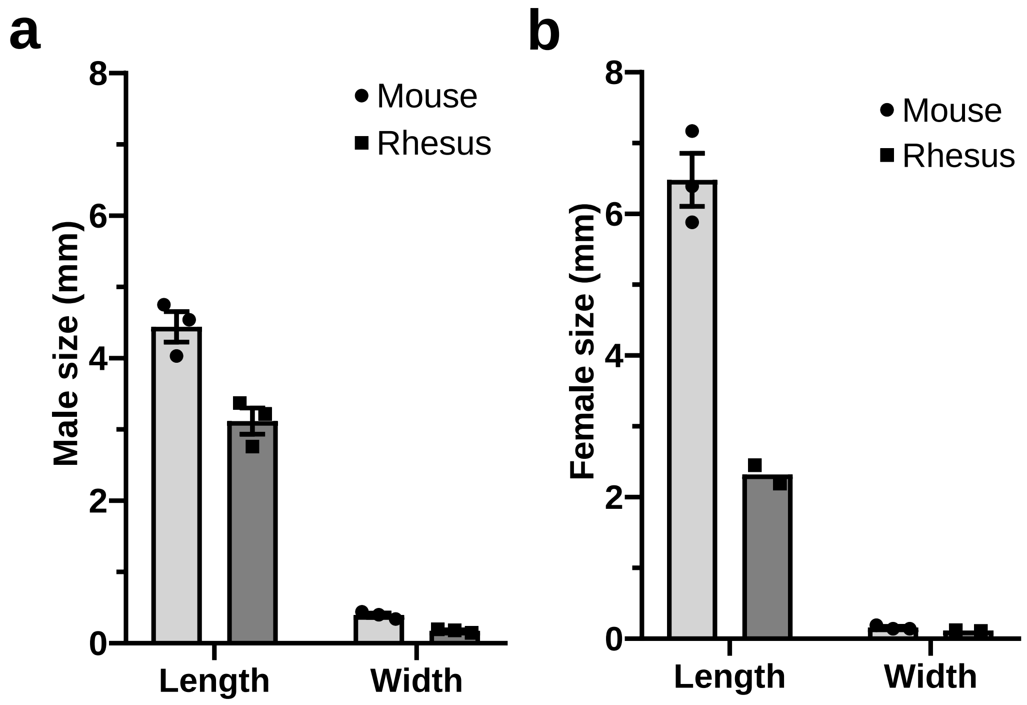

Supplementary Fig. 3. Worms recovered from rhesus macaques at W20pc are emaciated, compared to adults from permissive mice hosts. a Dimensions of three male worms recovered from rhesus macaques. **b** Dimensions of two female worms recovered from rhesus macaques. Three worms recovered from mice were used for comparison. For **a** and **b**, data are presented as mean values +/- SEM.

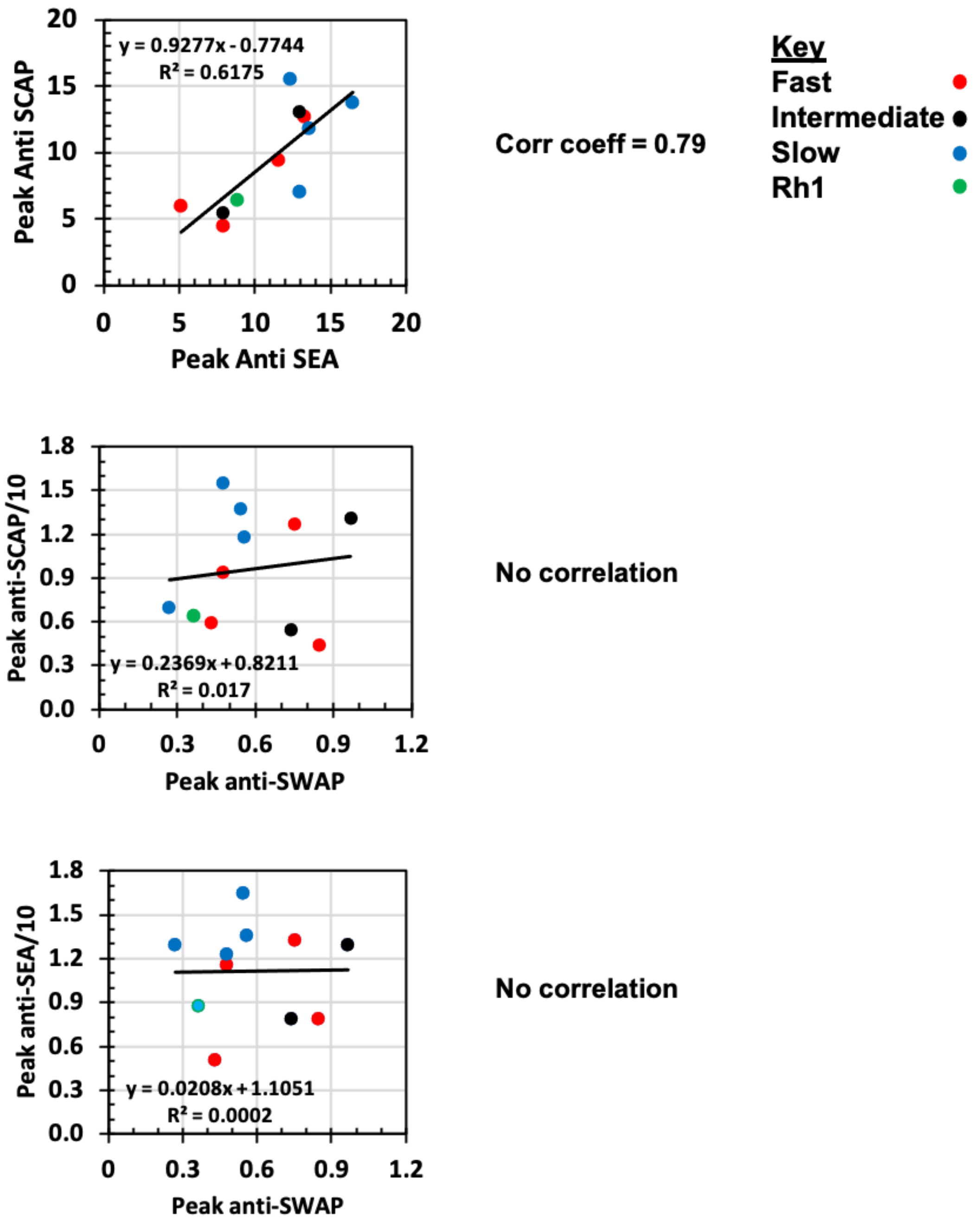

Supplementary Fig. 4. Peak SCAP (soluble cercarial antigen preparation) and SEA (soluble egg antigen) antibody responses correlate but other responses do not. a The maximum values attained by the responses to the soluble egg (SEA) and cercarial (SCAP) preparations correlate in individual macaques. R^2 for fit of regression = 0.62, Corr. Coeff. = 0.79. **b** The maximum values attained by the soluble adult worm (soluble antigen preparation of adult schistosomes, SWAP) and cercarial (SCAP) preparations do not correlate in individual macaques. R^2 for fit of regression = 0.017, Corr. Coeff. = NS. **c** The maximum values attained by the soluble adult worm (SWAP) and egg (SEA) preparations do not correlate in individual macaques. R^2 for fit of regression = 0.0002, Corr. Coeff. = NS.

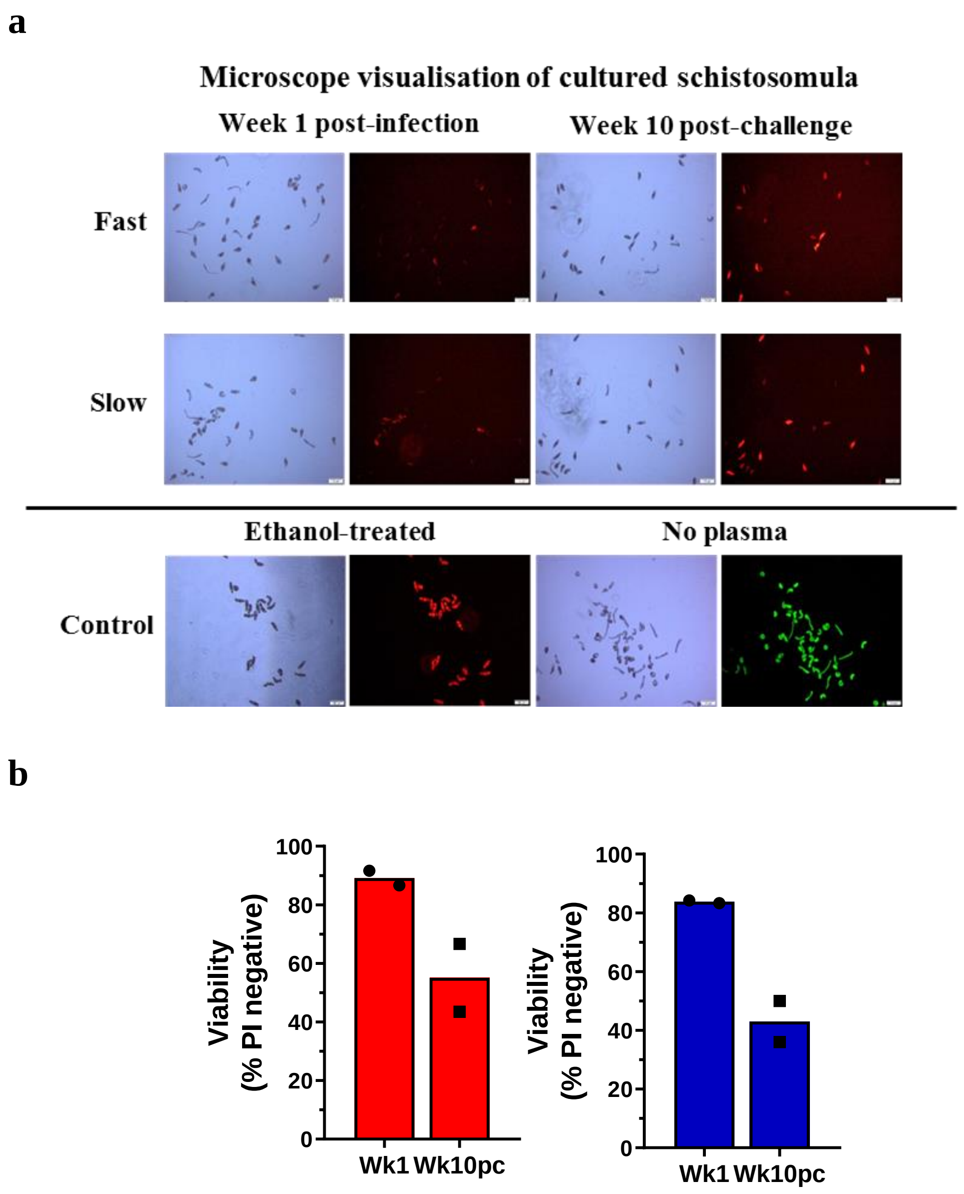

Supplementary Fig. 5. Microscope visualization of schistosomula cultured with rhesus plasma. a An additional assay was also used to visualise schistosomula viability by light microscopy. For this purpose, 3-day-old schistosomula were cultured for 48 h with a pool of rhesus plasma from Fast or Slow responders collected at Week 1 post-infection (Wk1) or Week 10 post-challenge (Wk10pc), and then stained with FDA/PI (Fluorescein diacetate/propidium iodide) before visual inspection and counting. Comparison of the brightfield versus fluorescent images revealed that PI-positive larvae (red) were more numerous in Wk10pc cultures than Wk1. The control larvae, cultured without addition of plasma, were 100% PI^+ (red) when ethanol-treated (when dead), but 100% viable in the presence of FDA^+ (green). Typical images from n = 2 biologically independent experiments. Magnification bars all at 200 µm. **b** Manual counting of PI^+ larvae at the end of the experiment revealed increased schistosomula killing by plasma collected at week 10 post-challenge (Wk10pc) when compared with plasma collected at week 1 post-infection (Wk1); pool of plasma from macaques of the Fast group (red bars) or the Slow group (blue bars). n = 2 biologically independent experiments.

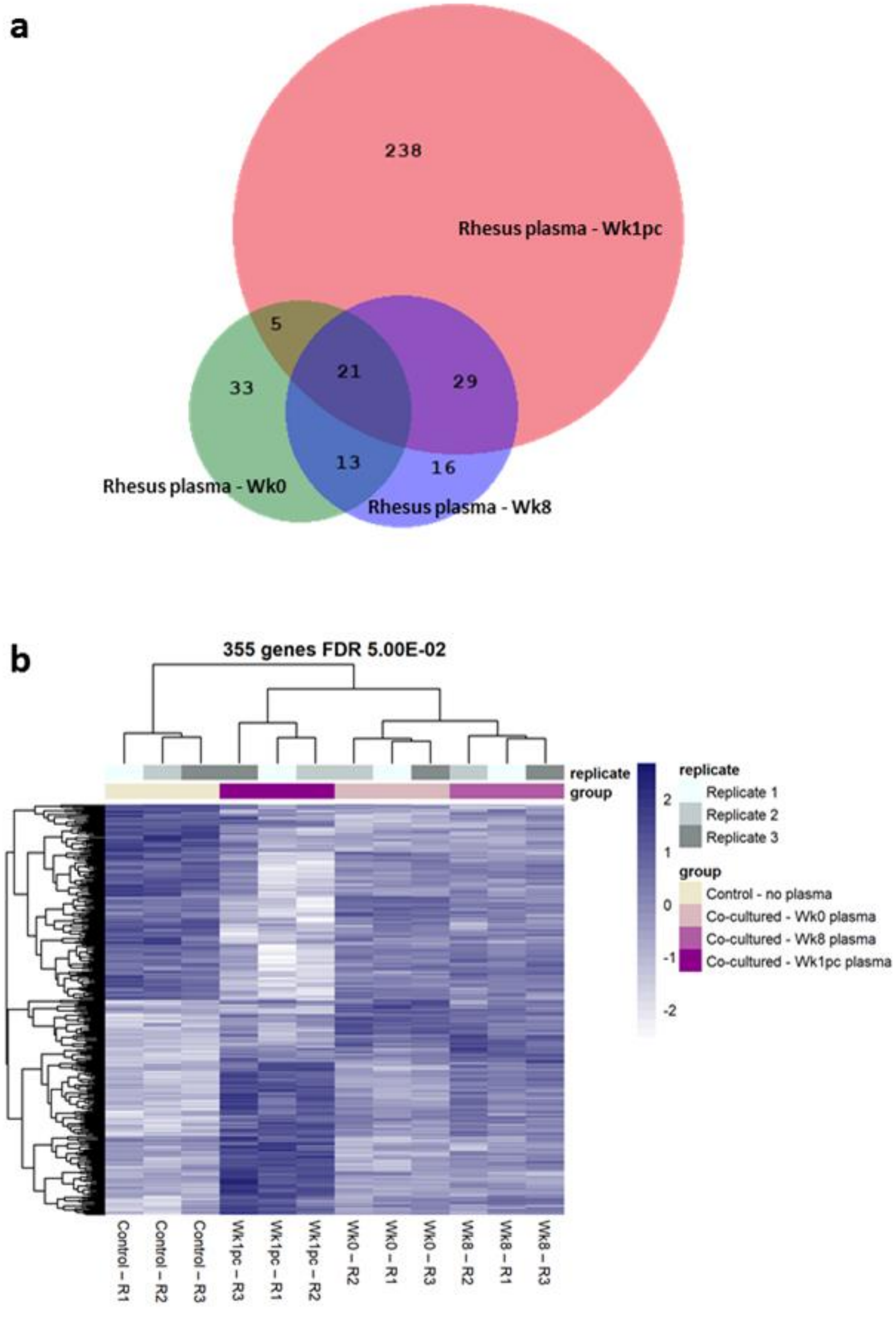

Supplementary Fig. 6. RNA-Seq analysis of *S. mansoni* schistosomula co-cultured *in vitro* with rhesus plasma. a Venn diagram showing the number of differentially expressed genes in each of the conditions (schistosomula co-cultured with rhesus plasma from Wk0, Wk8 or Wk1pc) compared with control schistosomula not exposed to plasma (FDR < 0.05). **b** Unsupervised heatmap clustering analysis showing the differentially expressed genes in *S. mansoni* schistosomula co-cultured with rhesus plasma collected at different weeks post-infection or post-challenge (FDR $\leq 5 \times 10^{-2}$). Each line represents a differentially expressed gene normalized by Z-score, according to the blue scale on the right. Each column represents one different sample and the colors at the top show the different biological replicates and type of treatment for each sample.

a

Estimation of antigen production by:
700 penetrant cercariae
160 adult pairs worm burden

Cercarial gland secretions §

0.24% of the cercarial body is released into the skin during penetration (the tail is lost)
The protein content of the schistosomulum body = 0.0363 µg
Total protein secreted into the skin = 0.0363 x 0.24 = 0.0087 µg x 700 = 6.1 µg

Vomitus *

12 µg of protein/day

Eggs †

Eggs/female/day = 300 x 160 = 48,000/day
Total soluble egg protein in mature eggs (- inert shell) = 168.5 µg/day.
Protein secreted by mature eggs in culture (96% viable) = 6.34 µg/day (3.76% of total protein)

Somatic antigens

Protein content per worm pair = 0.060 mg #
50% death of 160 worm pairs will release 4.8 mg protein into the bloodstream between wk 10 and 15

---

§ Supplementary ref. [2]
*** Dr Leandro Neves, personal communication**
† Dr William Mathieson, personal communication
# Supplementary ref. [3]

b

Prep	Stage	Antigen source	Signature constituents	Suppl. Ref.
SCAP	Cercaria	Acetabular glands	Elastases *, Metalloproteases, SmVALs, Sm16	4, 5
SSP	Migrating schistosomula	Head gland ‡<br>Tegument<br>Alimentary tract	MEG-2 and MEG-3 family<br>Membrane enzymes, Tetraspanins, Annexins<br>Esophageal gland MEGs and enzymes, Gastrodermal hydrolases, saposins	6, 7<br><br>8, 9
SEA §	Mature egg	Sub-shell envelope (live)<br>Contents (dead)	IPSE, Omega 1, MEG-2 and MEG-3 family<br>Total cytosol and cytoskeleton	10, 11<br>4
SWAP #	Adults	Tegument<br>Alimentary tract	Membrane enzymes, Tetraspanins, Annexins<br>Esophageal gland MEGs and enzymes, Gastrodermal hydrolases, saposins	12<br>8, 9

Footnotes

* elastases are known to be poorly immunogenic (Supplementary ref.[13])

‡ The MEG-3 and possibly the MEG-2 proteins may also be secreted by tegument homogeneous bodies

§ The highly immunogenic secretions of live eggs comprise a cohort of proteins integral to tissue migration (Supplementary ref. [10,14])

# SWAP is dominated by just 18 cytosolic and cytoskeletal proteins which make up 50% of the total content (Supplementary ref.[12]), an ideal indicator of worm death.

Supplementary Fig. 7. Estimation of antigen production by cercariae or adult worm pairs, and contents of soluble protein extract preparations. a Calculation of the amounts of proteins released into the skin by 700 penetrant cercariae, of the amounts of proteins in the parasites' vomitus, in the eggs laid per day by 160 females, and the amounts of proteins released by the 50% death of 160 worm pairs. **b** Description of the signature constituents of each of the four soluble protein extract preparations SCAP (soluble cercarial antigen preparation), SSP (soluble schistosomula protein), SEA

(soluble egg antigen), and SWAP (soluble antigen preparation of adult schistosomes), used in the ELISA immune assays.

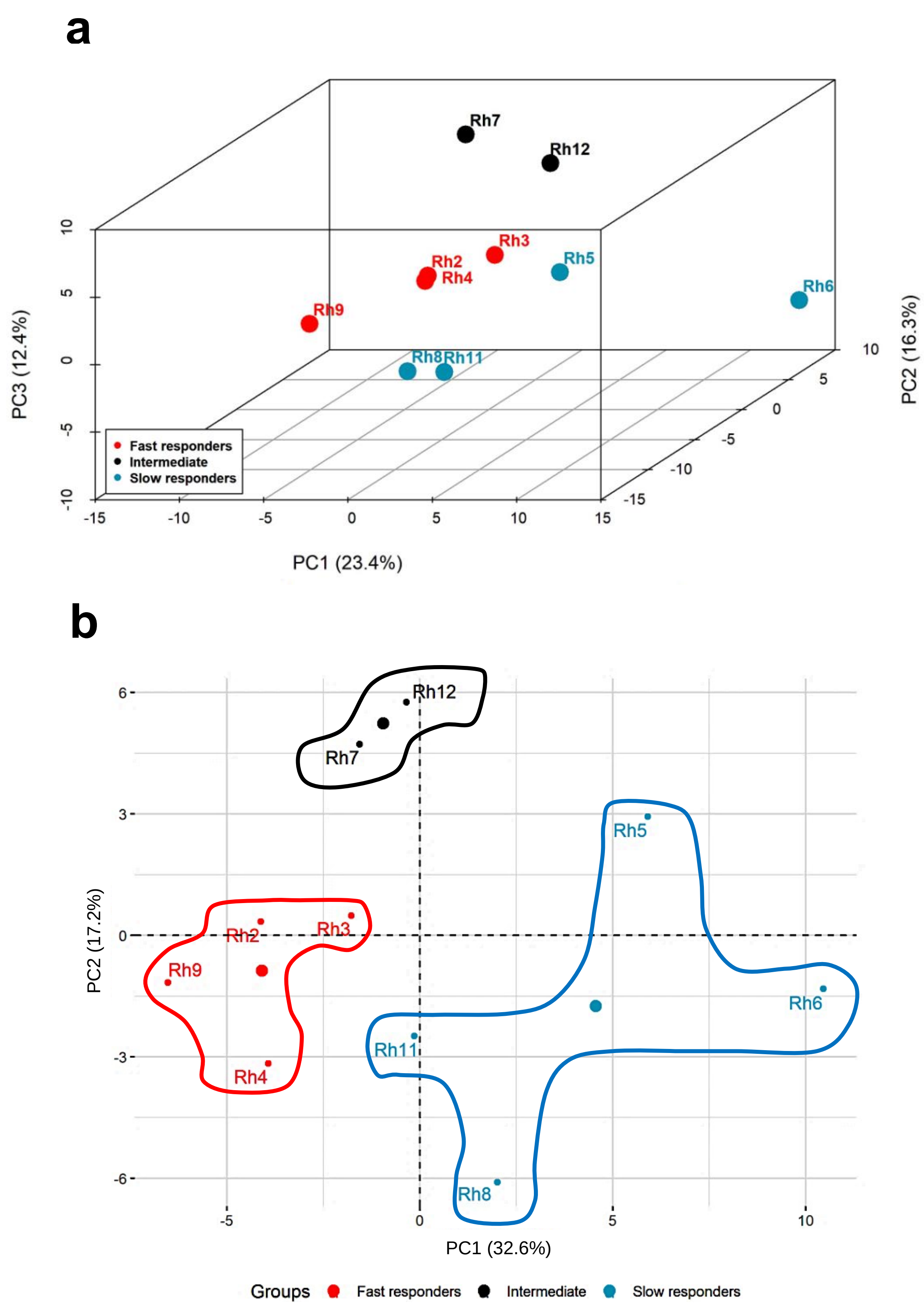

Supplementary Fig. 8. Principal components analysis. a Unsupervised clustering analysis using 2120 datapoints (212 events), distributed over all 15 observed parameters (see Methods), acquired over 62 weeks from 10 rhesus macaques. **b** Unsupervised clustering analysis using 780 datapoints (78 events), distributed over 5 selected parameters, namely CAA, EPG, anti-SCAP, anti-SEA, and anti-SWAP, acquired over 62 weeks from 10 rhesus macaques. In both analyses, less informative events were filtered

out (less informative events = those time points where 3 or more datapoints had zero value), representing 43 out of 255 events (17%) filtered out in (**a**) and 17 out of 95 events (18%) in (**b**). Each rhesus is coloured according to the Fast, Intermediate or Slow responder groups based on their cure rates (see Results). The larger symbols in (**b**) show the centroids of each of the three groups. The percent of total variance in the system explained by each principal component (PC1, PC2 and PC3 in (**a**) or PC1 and PC2 in (**b**)) is shown in the corresponding axis labels.

Supplementary references

1. Fallon PG, *et al.* Juvenile rhesus monkeys have lower type 2 cytokine responses than adults after primary infection with *Schistosoma mansoni*. *J Infect Dis* **187**, 939-945 (2003).
2. Harrop R, Wilson RA. Protein synthesis and release by cultured schistosomula of *Schistosoma mansoni*. *Parasitology* **107 (Pt 3)**, 265-274 (1993).
3. Mercer JG, Chappell LH. *Schistosoma mansoni*: effect of maintenance in vitro on the uptake and incorporation of leucine by adult worms. *Molecular and biochemical parasitology* **15**, 327-339 (1985).
4. Curwen RS, Ashton PD, Johnston DA, Wilson RA. The *Schistosoma mansoni* soluble proteome: a comparison across four life-cycle stages. *Molecular and biochemical parasitology* **138**, 57-66 (2004).
5. Curwen RS, Ashton PD, Sundaralingam S, Wilson RA. Identification of novel proteases and immunomodulators in the secretions of schistosome cercariae that facilitate host entry. *Molecular & cellular proteomics : MCP* **5**, 835-844 (2006).
6. Wangwiwatsin A, *et al.* Transcriptome of the parasitic flatworm *Schistosoma mansoni* during intra-mammalian development. *PLoS Negl Trop Dis* **14**, e0007743 (2020).
7. Wilson RA. Virulence factors of schistosomes. *Microbes and infection* **14**, 1442-1450 (2012).
8. Hall SL, Braschi S, Truscott M, Mathieson W, Cesari IM, Wilson RA. Insights into blood feeding by schistosomes from a proteomic analysis of worm vomitus. *Molecular and biochemical parasitology* **179**, 18-29 (2011).
9. Neves LX, *et al.* Quantitative Proteomics of Enriched Esophageal and Gut Tissues from the Human Blood Fluke *Schistosoma mansoni* Pinpoints Secreted Proteins for Vaccine Development. *Journal of proteome research* **19**, 314-326 (2020).
10. Schwartz C, Fallon PG. Schistosoma "Eggs-Iting" the Host: Granuloma Formation and Egg Excretion. *Frontiers in immunology* **9**, 2492 (2018).
11. Mathieson W, Wilson RA. A comparative proteomic study of the undeveloped and developed *Schistosoma mansoni* egg and its contents: the miracidium, hatch fluid and secretions. *Int J Parasitol* **40**, 617-628 (2010).
12. Neves LX, Sanson AL, Wilson RA, Castro-Borges W. What's in SWAP? Abundance of the principal constituents in a soluble extract of *Schistosoma mansoni* revealed by shotgun proteomics. *Parasit Vectors* **8**, 337 (2015).
13. Darani HY, Doenhoff MJ. Anomalous immunogenic properties of serine proteases. *Scandinavian journal of immunology* **70**, 384-388 (2009).
14. Kariuki TM, Farah IO, Wilson RA, Coulson PS. Antibodies elicited by the secretions from schistosome cercariae and eggs are predominantly against glycan epitopes. *Parasite Immunol* **30**, 554-562 (2008).
