# Supplementary material for: Rhesus macaques self-curing from a schistosome infection can display complete immunity to challenge
Source: Nat Commun. 2021 Oct 26;12:6181. doi: 10.1038/s41467-021-26497-0 (PMC8548296; doi:10.1038/s41467-021-26497-0)
Supplement: Supplementary file 3 — Description of Additional Supplementary Information [file 41467_2021_26497_MOESM3_ESM.docx]

**Description of Supplementary Data and Movie Files**

File Name: **Supplementary Data 1**
Description: **Table S1.** Parasitological and associated parameters from individual rhesus macaques.

File Name: **Supplementary Data 2**
Description: **Table S2.** Differential H3K4me3 abundance identified in the ChIP-Seq comparison: enriched genomic regions between schistosomula co-cultured with rhesus plasma collected at week 10 (Rhw10) and at week 0 (Rhw0)

File Name: **Supplementary Data 3**
Description: **Table S3.** Enriched Gene Ontology (GO) terms among genes presenting differential H3K4me3 marks abundance between schistosomula treated with rhesus plasma collected at week 10 pi (Rhw10) and at week 0 (Rhw0)

File Name: **Supplementary Data 4**
Description: **Table S4.** Differential H3K4me3 abundance identified in the ChIP-Seq comparison: enriched genomic regions between schistosomula co-cultured with rhesus plasma collected at week 10 pi (Rhw10) and at week 8 pi (Rhw8)

File Name: **Supplementary Data 5**
Description: **Table S5.** Enriched Gene Ontology (GO) terms among genes presenting differential H3K4me3 marks abundance between schistosomula co-cultured with rhesus plasma collected at week 8 pi (Rhw8) and at week 10 pi (Rhw10)

File Name: **Supplementary Data 6**Description: **Table S6.** Read count values for all genes in all samples, normalized across all conditions with “Trimmed Mean of M-values” (TMM) method.

File Name: **Supplementary Data 7**
Description: **Table S7.** Differentially expressed genes found with RNA-Seq in *S. mansoni* schistosomula co-cultured with plasma from week0, week8 and week1pc compared with control schistosomula

File Name: **Supplementary Data 8**
Description: **Table S8.** Enriched Gene Ontology (GO) terms among differentially expressed genes found with RNA-Seq in *S. mansoni* schistosomula co-cultured with plasma from week1pc compared with non-treated schistosomula

File Name: **Supplementary Data 9**
Description: **Table S9.** Genes of the early autophagy pathway

File Name: **Supplementary Data 10**
Description: **Table S10.** Summary of ChIP-Seq reads filtering and alignment

File Name: **Supplementary Data 11**
Description: **Table S11.** Summary of ChIP-Seq peaks identified

File Name: **Supplementary Data 12**
Description: **Table S12.** Summary of *S. mansoni* schistosomula RNA-Seq reads filtering and alignment

File Name: **Supplementary Movie 1**
Description: **Movie 1.** Motility of 3-day-old schistosomula after co-culture for 48 h with rhesus plasma collected at Wk16 post-infection

File Name: **Supplementary Movie 2**
Description: **Movie 2.** Motility of 3-day-old schistosomula after co-culture for 48 h with rhesus plasma collected at Wk20 post-challenge (Wk62)

File Name: **Supplementary Movie 3**
Description: **Movie 3.** Motility of control 3-day-old schistosomula after a further 48 h in culture
