# Supplementary material for: Rhesus macaques self-curing from a schistosome infection can display complete immunity to challenge
Source: Nat Commun. 2021 Oct 26;12:6181. doi: 10.1038/s41467-021-26497-0 (PMC8548296; doi:10.1038/s41467-021-26497-0)
Supplement: Supplementary file 19 — Reporting Summary [file 41467_2021_26497_MOESM19_ESM.pdf]

nature research

Corresponding author(s): Prof. Dr. Sergio Verjovski-Almeida

Last updated by author(s): September 23, 2021

Reporting Summary

Nature Research wishes to improve the reproducibility of the work that we publish. This form provides structure for consistency and transparency in reporting. For further information on Nature Research policies, see our Editorial Policies and the Editorial Policy Checklist.

Statistics

For all statistical analyses, confirm that the following items are present in the figure legend, table legend, main text, or Methods section.

n/a	Confirmed	
☐	☒	The exact sample size (*n*) for each experimental group/condition, given as a discrete number and unit of measurement
☐	☒	A statement on whether measurements were taken from distinct samples or whether the same sample was measured repeatedly
☐	☒	The statistical test(s) used AND whether they are one- or two-sided<br>*Only common tests should be described solely by name; describe more complex techniques in the Methods section.*
☒	☐	A description of all covariates tested
☒	☐	A description of any assumptions or corrections, such as tests of normality and adjustment for multiple comparisons
☐	☒	A full description of the statistical parameters including central tendency (e.g. means) or other basic estimates (e.g. regression coefficient) AND variation (e.g. standard deviation) or associated estimates of uncertainty (e.g. confidence intervals)
☐	☒	For null hypothesis testing, the test statistic (e.g. *F*, *t*, *r*) with confidence intervals, effect sizes, degrees of freedom and *P* value noted<br>*Give P values as exact values whenever suitable.*
☒	☐	For Bayesian analysis, information on the choice of priors and Markov chain Monte Carlo settings
☒	☐	For hierarchical and complex designs, identification of the appropriate level for tests and full reporting of outcomes
☒	☐	Estimates of effect sizes (e.g. Cohen's *d*, Pearson's *r*), indicating how they were calculated

Our web collection on statistics for biologists contains articles on many of the points above.

Software and code

Policy information about availability of computer code

Data collection	CAA quantitation, Anti-SEA and anti-SCAP detection, IP-10 and SAA quantitation: Fluorocount microplate reader (Packard). Anti-SWAP and anti-SSP ELISA: Spectramax Paradigm (Molecular Devices). Cell counting: ABX Pentra XL 80 instrument (Horiba). Morphological analysis of recovered worms: LSM710 confocal microscope (Zeiss). Schistosomula images: CKX41/U-RFLT50 inverted microscope (Olympus). ChIP-Seq libraries electrophoresis: 2100 Bioanalyzer (Agilent Technologies). ChIP-Seq libraries sequencing: NextSeq 550 instrument (Illumina). RNA-Seq data acquisition: Stranded tagged cDNA libraries were prepared by BGI Genomics using the Strand-Specific Transcriptome Library Construction Protocol (DNBSEQ, SOP-SS-115) and quantification and quality were performed on a 2100 Bioanalyzer (Agilent). Libraries were pooled and sequenced (300 cycles, paired-end sequencing) on a MGISEQ-2000 instrument (BGI Genomics).
Data analysis	ChIP-Seq analysis: Fastp (v0.20.0), bowtie2 (v.2.2.9), Samtools (v.1.8), picard-tools MarkDuplicates (v.1.95), Qualimap (v.2.2.1), MACS2 (v2.1.1) using the AQUAS pipeline (https://github.com/NHLBI-BCB/TF_chipseq_pipeline), DiffBind (2.12.0), BINGO (3.0.2). Other statistical analyses: GraphPad Prism v.8.0; Solver tool in Microsoft Excel (version 15.0.5371.1000) to find the best fit to the logistic equation for the CAA data from week 1 to week 10. RNA-Seq analysis: RNA-Seq reads were mapped using STAR (v 2.7.3a). Read counting was performed with RSEM (v1.3.1). Read count values are shown as log2CPM normalized across all conditions with "Trimmed Mean of M-values" (TMM) method implemented in the edgeR package within the R platform (v 3.6.2). Genewise dispersion estimation was calculated with edgeR (v 3.30.3) using all twelve samples, and statistical analysis for identification of differentially expressed genes (DEGs) was performed by comparing each co-cultured group to control with two different algorithms: (i) limma+voom (v 3.44.3) and (ii) edgeR+svaseq (v 3.30.3). The DEGs are shown with an unsupervised heatmap clustering that was built using the pheatmap (v 1.0.12) function in the R platform (v 3.6.2).

For manuscripts utilizing custom algorithms or software that are central to the research but not yet described in published literature, software must be made available to editors and reviewers. We strongly encourage code deposition in a community repository (e.g. GitHub). See the Nature Research guidelines for submitting code & software for further information.

Data

Policy information about availability of data

All manuscripts must include a data availability statement. This statement should provide the following information, where applicable:

- Accession codes, unique identifiers, or web links for publicly available datasets
- A list of figures that have associated raw data
- A description of any restrictions on data availability

All ChIP-seq and RNA-seq data generated in this study were deposited in the NCBI Sequence Read Archive (http://www.ncbi.nlm.nih.gov/sra) under BioProject ID PRJNA602708 (https://www.ncbi.nlm.nih.gov/Traces/study/?acc=PRJNA602708&o=acc_s%3Aa). The authors declare that all other data supporting the findings of this study are accessible within the article and its Supplementary Information files. Source data are provided with this paper as a Source Data file.

Field-specific reporting

Please select the one below that is the best fit for your research. If you are not sure, read the appropriate sections before making your selection.

☒ Life sciences ☐ Behavioural & social sciences ☐ Ecological, evolutionary & environmental sciences

For a reference copy of the document with all sections, see nature.com/documents/nr-reporting-summary-flat.pdf

Life sciences study design

All studies must disclose on these points even when the disclosure is negative.

Sample size	Rhesus macaques: we infected and challenged 12 rhesus macaques with 700 cercariae of Schistosoma mansoni. Rhesus macaques are outbred animals, and previous experiments (Wilson et al., 2008 and Li et al., 2015) have suggested a wide range of responses among these animals. To increase the statistical power of our analysis, we decided to increase the number of rhesus macaques compared to previous works (both Wilson et al., 2008 and Li et al., 2015 studies used 6 rhesus macaques). We also followed the recommendations of the Weatherall report (2006) on "The use of non-human primates in research" and the UK NC3Rs Guidelines "Primate accommodation, care and use (revised version, October 2017)", which support the replacement, refinement and reduction of primate use in research.
Data exclusions	Rhesus10 became unwell after Wk10 and was withdrawn from the study at that point. The CAA data collected from Rhesus 10 up to Wk10 were used to estimate Rh10 worm burden, and no other data from Rh10 were used in further analyses.
Replication	In vitro schistosomula treatment: we performed three biological replicates for the ATP assays, each containing schistosomula obtained from cercariae from different batches of infected snails, and each biological replicate included two technical replicates, each processed separately but in parallel. For the PI/FDA staining and ChIP-Seq assays, two biological replicates were performed. For the RNA-Seq assays, three biological replicates were performed.This information is described in the methods, the text and figure captions. Experimental findings were reliably reproduced by technical and biological replicates.CAA quantitation: one technical replicate. EPG: three technical replicates. Anti-SEA and anti-SCAP quantitation: two technical replicates. Anti-SWAP and anti-SPP quantitation: two technical replicates. Haematology, IP-10 and SAA quantitation, confocal microscopy: one technical replicate.
Randomization	Randomization was not performed in the rhesus macaque population, since this study assessed changes after infection and after challenge in all individuals within the same experimental group.
Blinding	Blinding was not performed since all study rhesus macaques belonged to one experimental group.

Behavioural & social sciences study design

All studies must disclose on these points even when the disclosure is negative.

Study description	*Briefly describe the study type including whether data are quantitative, qualitative, or mixed-methods (e.g. qualitative cross-sectional, quantitative experimental, mixed-methods case study).*
Research sample	*State the research sample (e.g. Harvard university undergraduates, villagers in rural India) and provide relevant demographic information (e.g. age, sex) and indicate whether the sample is representative. Provide a rationale for the study sample chosen. For studies involving existing datasets, please describe the dataset and source.*
Sampling strategy	*Describe the sampling procedure (e.g. random, snowball, stratified, convenience). Describe the statistical methods that were used to predetermine sample size OR if no sample-size calculation was performed, describe how sample sizes were chosen and provide a rationale for why these sample sizes are sufficient. For qualitative data, please indicate whether data saturation was considered, and what criteria were used to decide that no further sampling was needed.*
Data collection	*Provide details about the data collection procedure, including the instruments or devices used to record the data (e.g. pen and paper, computer, eye tracker, video or audio equipment) whether anyone was present besides the participant(s) and the researcher, and whether the researcher was blind to experimental condition and/or the study hypothesis during data collection.*

Timing	*Indicate the start and stop dates of data collection. If there is a gap between collection periods, state the dates for each sample cohort.*
Data exclusions	*If no data were excluded from the analyses, state so OR if data were excluded, provide the exact number of exclusions and the rationale behind them, indicating whether exclusion criteria were pre-established.*
Non-participation	*State how many participants dropped out/declined participation and the reason(s) given OR provide response rate OR state that no participants dropped out/declined participation.*
Randomization	*If participants were not allocated into experimental groups, state so OR describe how participants were allocated to groups, and if allocation was not random, describe how covariates were controlled.*

Ecological, evolutionary & environmental sciences study design

All studies must disclose on these points even when the disclosure is negative.

Study description	*Briefly describe the study. For quantitative data include treatment factors and interactions, design structure (e.g. factorial, nested, hierarchical), nature and number of experimental units and replicates.*
Research sample	*Describe the research sample (e.g. a group of tagged Passer domesticus, all Stenocereus thurberi within Organ Pipe Cactus National Monument), and provide a rationale for the sample choice. When relevant, describe the organism taxa, source, sex, age range and any manipulations. State what population the sample is meant to represent when applicable. For studies involving existing datasets, describe the data and its source.*
Sampling strategy	*Note the sampling procedure. Describe the statistical methods that were used to predetermine sample size OR if no sample-size calculation was performed, describe how sample sizes were chosen and provide a rationale for why these sample sizes are sufficient.*
Data collection	*Describe the data collection procedure, including who recorded the data and how.*
Timing and spatial scale	*Indicate the start and stop dates of data collection, noting the frequency and periodicity of sampling and providing a rationale for these choices. If there is a gap between collection periods, state the dates for each sample cohort. Specify the spatial scale from which the data are taken*
Data exclusions	*If no data were excluded from the analyses, state so OR if data were excluded, describe the exclusions and the rationale behind them, indicating whether exclusion criteria were pre-established.*
Reproducibility	*Describe the measures taken to verify the reproducibility of experimental findings. For each experiment, note whether any attempts to repeat the experiment failed OR state that all attempts to repeat the experiment were successful.*
Randomization	*Describe how samples/organisms/participants were allocated into groups. If allocation was not random, describe how covariates were controlled. If this is not relevant to your study, explain why.*
Blinding	*Describe the extent of blinding used during data acquisition and analysis. If blinding was not possible, describe why OR explain why blinding was not relevant to your study.*

Did the study involve field work? ☐ Yes ☐ No

Field work, collection and transport

Field conditions	*Describe the study conditions for field work, providing relevant parameters (e.g. temperature, rainfall).*
Location	*State the location of the sampling or experiment, providing relevant parameters (e.g. latitude and longitude, elevation, water depth).*
Access & import/export	*Describe the efforts you have made to access habitats and to collect and import/export your samples in a responsible manner and in compliance with local, national and international laws, noting any permits that were obtained (give the name of the issuing authority, the date of issue, and any identifying information).*
Disturbance	*Describe any disturbance caused by the study and how it was minimized.*

Reporting for specific materials, systems and methods

We require information from authors about some types of materials, experimental systems and methods used in many studies. Here, indicate whether each material, system or method listed is relevant to your study. If you are not sure if a list item applies to your research, read the appropriate section before selecting a response.

Materials & experimental systems

n/a	Involved in the study	
	☒	Antibodies
☒		Eukaryotic cell lines
☒		Palaeontology and archaeology
	☒	Animals and other organisms
☒		Human research participants
☒		Clinical data
☒		Dual use research of concern

Methods

n/a	Involved in the study	
	☒	ChIP-seq
☒		Flow cytometry
☒		MRI-based neuroimaging

Antibodies

Antibodies used

Anti-H3K4me3 antibody (Diagenode C15410003; lot A1051D and A1052D);
anti-CAA antibody developed at the laboratory of Dr. Paul L.A.M. Corstjens, mouse monoclonal, anti-CAA clone 147 (LUMC, Parasitology); a sandwich with the same antibody on the LF strip and the UCP.
Mouse monoclonal for SAA sandwich: on the LF strip mAb anti-SAA1 clone SAA15 [NB100-73077], the UCP conjugate with mAb clone SAA1 [NB100-73071]; both from Novus Biologicals, Littleton, USA
Mouse monoclonal for IP-10 sandwich: on the LF strip mAb anti-IP10 clone B-C55 [879.950], the UCP conjugate with mAb clone B-C50 [855.420]; both from Diaclone Research, Besancon, France

Validation

Anti-H3K4me3 antibody (Diagenode C15410003; lot A1051D and A1052D) has been previously tested for specificity and shown to be suitable for ChIP-Seq with Schistosoma mansoni (doi: https://doi.org/10.1371/journal.ppat.1007066).
Anti-CAA antibody (anti-CAA clone 147, LUMC, Parasitology) has been validated at Corstjens PL, et al. Tools for diagnosis, monitoring and screening of Schistosoma infections utilizing lateral-flow based assays and upconverting phosphor labels. Parasitology 141, 1841-1855 (2014). https://doi.org/10.1017/S0031182014000626
The lateral flow test for detection of IP-10 in blood samples with the use of the anti-IP10 antibody was validated at https://doi.org/10.1016/j.clinbiochem.2015.08.013
The lateral flow test for detection of SAA in blood samples with the use of anti-SAA antibody was validated in humans, i.e. in TB vs. ORD (other respiratory disease) and COVID patients/suspects (unpublished data).

Eukaryotic cell lines

Policy information about cell lines

Cell line source(s)

State the source of each cell line used.

Authentication

Describe the authentication procedures for each cell line used OR declare that none of the cell lines used were authenticated.

Mycoplasma contamination

Confirm that all cell lines tested negative for mycoplasma contamination OR describe the results of the testing for mycoplasma contamination OR declare that the cell lines were not tested for mycoplasma contamination.

Commonly misidentified lines
(See ICLAC register)

Name any commonly misidentified cell lines used in the study and provide a rationale for their use.

Palaeontology and Archaeology

Specimen provenance

Provide provenance information for specimens and describe permits that were obtained for the work (including the name of the issuing authority, the date of issue, and any identifying information).

Specimen deposition

Indicate where the specimens have been deposited to permit free access by other researchers.

Dating methods

If new dates are provided, describe how they were obtained (e.g. collection, storage, sample pretreatment and measurement), where they were obtained (i.e. lab name), the calibration program and the protocol for quality assurance OR state that no new dates are provided.

☐ Tick this box to confirm that the raw and calibrated dates are available in the paper or in Supplementary Information.

Ethics oversight

Identify the organization(s) that approved or provided guidance on the study protocol, OR state that no ethical approval or guidance was required and explain why not.

Note that full information on the approval of the study protocol must also be provided in the manuscript.

Animals and other organisms

Policy information about studies involving animals; ARRIVE guidelines recommended for reporting animal research

Laboratory animals	- The complete Schistosoma mansoni life cycle is maintained at Instituto Butantan.<br>- Schistosoma mansoni: strain BH (mixed sexes)<br>- Golden Hamster host: Mesocricetus auratus (females, age 21 days) - approved by the Ethics Committee for Animal Experimentation of Butantan Institute (CEUAIB n˚ 6748040515).<br>- Rhesus macaque: Macaca mulatta (females, mean age 13.9 ± 2.8 years) – 12 animals from a colony maintained at Butantan Institute since 1929, which was started for the purpose of studying Yellow Fever virus vaccine – Experimental design for the present Schistosoma mansoni study approved by the Ethics Committee for Animal Experimentation of Butantan Institute (CEUAIB n˚ 1388/ 15).<br>- Mouse host - Mus musculus (females, aged 35 days) - experimentation with mice followed the recommendations from the Biology Department Ethics Committee, University of York, and experiments were performed on personal (PIL 50/592) and project licences (PPL 60/4340) issued to RAW.
Wild animals	This study did not involve wild animals
Field-collected samples	This study did not involve samples collected from the field
Ethics oversight	- Housing conditions of the rhesus macaques and experimental protocols used in the study were in strict accordance with the Ethical Principles in Animal Research adopted by the Conselho Nacional de Controle de Experimentação Animal (CONCEA) and were approved by the Institutional Animal Care and Use Committee of Instituto Butantan (CEUAIB 1388/ 15). The study was carried out in compliance with the ARRIVE guidelines. Design and execution of the study complied with the recommendations of the Weatherall report (2006) and with principles set out in the UK NC3Rs Guidelines “Primate accommodation, care and use (revised version, October 2017)” (http://www.nc3rs.org.uk/primatesguidelines).<br>- Housing conditions of the hamsters and experimental procedures used in this study were also in strict accordance with the Ethical Principles in Animal Research adopted by the CONCEA and the experimental protocol was approved by the Ethics Committee for Animal Experimentation of Butantan Institute (CEUAIB n˚ 6748040515).<br>- Housing conditions and experimentation with mice followed the recommendations from the Biology Department Ethics Committee, University of York, and experiments were performed on personal (PIL 50/592) and project licences (PPL 60/4340) issued to RAW.

Note that full information on the approval of the study protocol must also be provided in the manuscript.

Human research participants

Policy information about studies involving human research participants

Population characteristics	*Describe the covariate-relevant population characteristics of the human research participants (e.g. age, gender, genotypic information, past and current diagnosis and treatment categories). If you filled out the behavioural & social sciences study design questions and have nothing to add here, write "See above."*
Recruitment	*Describe how participants were recruited. Outline any potential self-selection bias or other biases that may be present and how these are likely to impact results.*
Ethics oversight	*Identify the organization(s) that approved the study protocol.*

Note that full information on the approval of the study protocol must also be provided in the manuscript.

Clinical data

Policy information about clinical studies

All manuscripts should comply with the ICMJEguidelines for publication of clinical research and a completedCONSORT checklist must be included with all submissions.

Clinical trial registration	*Provide the trial registration number from ClinicalTrials.gov or an equivalent agency.*
Study protocol	*Note where the full trial protocol can be accessed OR if not available, explain why.*
Data collection	*Describe the settings and locales of data collection, noting the time periods of recruitment and data collection.*
Outcomes	*Describe how you pre-defined primary and secondary outcome measures and how you assessed these measures.*

Dual use research of concern

Policy information about dual use research of concern

Hazards

Could the accidental, deliberate or reckless misuse of agents or technologies generated in the work, or the application of information presented in the manuscript, pose a threat to:

No	Yes	
[x]	[ ]	Public health
[x]	[ ]	National security
[x]	[ ]	Crops and/or livestock
[x]	[ ]	Ecosystems
[x]	[ ]	Any other significant area

Experiments of concern

Does the work involve any of these experiments of concern:

No	Yes	
[x]	[ ]	Demonstrate how to render a vaccine ineffective
[x]	[ ]	Confer resistance to therapeutically useful antibiotics or antiviral agents
[x]	[ ]	Enhance the virulence of a pathogen or render a nonpathogen virulent
[x]	[ ]	Increase transmissibility of a pathogen
[x]	[ ]	Alter the host range of a pathogen
[x]	[ ]	Enable evasion of diagnostic/detection modalities
[x]	[ ]	Enable the weaponization of a biological agent or toxin
[x]	[ ]	Any other potentially harmful combination of experiments and agents

ChIP-seq

Data deposition

[x] Confirm that both raw and final processed data have been deposited in a public database such as GEO.

[x] Confirm that you have deposited or provided access to graph files (e.g. BED files) for the called peaks.

Data access links<br>*May remain private before publication.*	https://www.ncbi.nlm.nih.gov/sra/?term=PRJNA602708
Files in database submission	H3K4me3Rh0w-biological replicate1<br>H3K4me3Rh0w-biological replicate2<br>H3K4me3Rh8w-biological replicate1<br>H3K4me3Rh8w-biological replicate2<br>H3K4me3Rh10w-biological replicate1<br>H3K4me3Rh10w-biological replicate2
Genome browser session<br>(e.g. UCSC)	http://schistosoma.usp.br<br>http://genome.verjolab.usp.br/cgi-bin/hgTracks?db=hub_127_schMan3&position=SM_V7_4%<br>3A33471205-33483068&hgsid=158749_2jd2GW5W6EjFusNICs3YIRaNe4MO

Methodology

Replicates	Two biological replicates were assayed, each containing schistosomula obtained from cercariae from different batches of infected snails.
Sequencing depth	These data are explicitly described in Supplementary Data 10.
Antibodies	Anti-H3K4me3 (Diagenode C15410003; lot A1051D and A1052D)
Peak calling parameters	peak calling was performed with MACS2 (v2.1.1) using the AQUAS pipeline (https://github.com/NHLBI-BCB/TF_chipseq_pipeline).
Data quality	Quality check of reads was performed using FastQC (v.0.11.7, https://www.bioinformatics.babraham.ac.uk/projects/fastqc/). Fastp (v0.20.0) was used to trim adapters and reads with low sequencing quality. Peak analyses showed that on average ~2500 peaks were identified per sample with an average peak length of 170 bp (Supplementary Data 11), at a significance threshold p-value of 0.01.
Software	Quality check of reads was performed using FastQC (v.0.11.7, https://www.bioinformatics.babraham.ac.uk/projects/fastqc/). Fastp (v0.20.0) was used to trim adapters and reads with low sequencing quality. Reads were mapped using bowtie2 (v.2.2.9) against the S. mansoni genome PRJEA36577 (v7) retrieved from WormBase (schistosoma_mansoni.PRJEA36577.WBPS14.genomic_softmasked.fa) and the overall average mapping rate of ChIP-Seq reads to the genome was 93% (Supplementary Data 10). Default parameters were used to report only the best alignment of each paired-end read. Samtools (v.1.8) and picard-tools MarkDuplicates (v.1.95) (https://broadinstitute.github.io/picard/) were used to filter and remove PCR and optical duplications; filtering resulted in ~58% of mapped reads remaining for further analysis (Supplementary Data 10). Qualimap (v.2.2.1) was used for mapping quality control. After removal of reads mapping to mitochondria, peak calling was

performed with MACS2 (v2.1.1) using the AQUAS pipeline (https://github.com/NHLBI-BCB/TF_chipseq_pipeline).

Flow Cytometry

Plots

Confirm that:

☐ The axis labels state the marker and fluorochrome used (e.g. CD4-FITC).

☐ The axis scales are clearly visible. Include numbers along axes only for bottom left plot of group (a 'group' is an analysis of identical markers).

☐ All plots are contour plots with outliers or pseudocolor plots.

☐ A numerical value for number of cells or percentage (with statistics) is provided.

Methodology

Sample preparation	*Describe the sample preparation, detailing the biological source of the cells and any tissue processing steps used.*
Instrument	*Identify the instrument used for data collection, specifying make and model number.*
Software	*Describe the software used to collect and analyze the flow cytometry data. For custom code that has been deposited into a community repository, provide accession details.*
Cell population abundance	*Describe the abundance of the relevant cell populations within post-sort fractions, providing details on the purity of the samples and how it was determined.*
Gating strategy	*Describe the gating strategy used for all relevant experiments, specifying the preliminary FSC/SSC gates of the starting cell population, indicating where boundaries between "positive" and "negative" staining cell populations are defined.*

☐ Tick this box to confirm that a figure exemplifying the gating strategy is provided in the Supplementary Information.

Magnetic resonance imaging

Experimental design

Design type	*Indicate task or resting state; event-related or block design.*
Design specifications	*Specify the number of blocks, trials or experimental units per session and/or subject, and specify the length of each trial or block (if trials are blocked) and interval between trials.*
Behavioral performance measures	*State number and/or type of variables recorded (e.g. correct button press, response time) and what statistics were used to establish that the subjects were performing the task as expected (e.g. mean, range, and/or standard deviation across subjects).*

Acquisition

Imaging type(s)	*Specify: functional, structural, diffusion, perfusion.*
Field strength	*Specify in Tesla*
Sequence & imaging parameters	*Specify the pulse sequence type (gradient echo, spin echo, etc.), imaging type (EPI, spiral, etc.), field of view, matrix size, slice thickness, orientation and TE/TR/flip angle.*
Area of acquisition	*State whether a whole brain scan was used OR define the area of acquisition, describing how the region was determined.*

Diffusion MRI ☐ Used ☐ Not used

Preprocessing

Preprocessing software	*Provide detail on software version and revision number and on specific parameters (model/functions, brain extraction, segmentation, smoothing kernel size, etc.).*
Normalization	*If data were normalized/standardized, describe the approach(es): specify linear or non-linear and define image types used for transformation OR indicate that data were not normalized and explain rationale for lack of normalization.*
Normalization template	*Describe the template used for normalization/transformation, specifying subject space or group standardized space (e.g. original Talairach, MNI305, ICBM152) OR indicate that the data were not normalized.*
Noise and artifact removal	*Describe your procedure(s) for artifact and structured noise removal, specifying motion parameters, tissue signals and physiological signals (heart rate, respiration).*

Volume censoring | *Define your software and/or method and criteria for volume censoring, and state the extent of such censoring.*

Statistical modeling & inference

Model type and settings | *Specify type (mass univariate, multivariate, RSA, predictive, etc.) and describe essential details of the model at the first and second levels (e.g. fixed, random or mixed effects; drift or auto-correlation).*

Effect(s) tested | *Define precise effect in terms of the task or stimulus conditions instead of psychological concepts and indicate whether ANOVA or factorial designs were used.*

Specify type of analysis: ☐ Whole brain ☐ ROI-based ☐ Both

Statistic type for inference (See Eklund et al. 2016) | *Specify voxel-wise or cluster-wise and report all relevant parameters for cluster-wise methods.*

Correction | *Describe the type of correction and how it is obtained for multiple comparisons (e.g. FWE, FDR, permutation or Monte Carlo).*

Models & analysis

n/a	Involved in the study
☐	☐ Functional and/or effective connectivity
☐	☐ Graph analysis
☐	☐ Multivariate modeling or predictive analysis

Functional and/or effective connectivity | *Report the measures of dependence used and the model details (e.g. Pearson correlation, partial correlation, mutual information).*

Graph analysis | *Report the dependent variable and connectivity measure, specifying weighted graph or binarized graph, subject- or group-level, and the global and/or node summaries used (e.g. clustering coefficient, efficiency, etc.).*

Multivariate modeling and predictive analysis | *Specify independent variables, features extraction and dimension reduction, model, training and evaluation metrics.*
